# Focused Review of Perioperative Care of Patients with Pulmonary Hypertension and Proposal of a Perioperative Pathway

**DOI:** 10.7759/cureus.2072

**Published:** 2018-01-15

**Authors:** Jochen Steppan, Natalia Diaz-Rodriguez, Viachaslau M Barodka, Daniel Nyhan, Erica Pullins, Traci Housten, Rachel L Damico, Stephen C Mathai, Paul M Hassoun, Dan E Berkowitz, Bryan G Maxwell, Todd M Kolb

**Affiliations:** 1 Anesthesiology and Critical Care Medicine, The Johns Hopkins University School of Medicine; 2 Anesthesia and Critical Care Medicine, The Johns Hopkins University School of Medicine; 3 Division of Pulmonary and Critical Care Medicine, The Johns Hopkins University School of Medicine; 4 Oregon Anesthesiology Group Pc, Legacy Health

**Keywords:** pulmonary hypertension, perioperative management, surgical home, high risk surgery, pulmonary arterial hypertension

## Abstract

Morbidity and mortality risk increase considerably for patients with pulmonary hypertension (PH) undergoing non-cardiac surgery. Unfortunately, there are no comprehensive, evidence-based guidelines for perioperative evaluation and management of these patients. We present a brief review of the literature on perioperative outcomes for patients with PH and describe the implementation of a collaborative perioperative management program for these high-risk patients at a tertiary academic center.

## Introduction and background

Patients with pulmonary hypertension (PH) present a unique and increasingly common challenge to the perioperative physician. Evolving demographics [1] and improving survival in the modern treatment era [2] for patients with pulmonary arterial hypertension (PAH), who often have the most severe hemodynamic derangements, have contributed to increasing numbers of these complex patients presenting for non-cardiac surgery. However, there are no comprehensive, evidence-based guidelines for perioperative management of patients with PH, despite their increased risk for perioperative complications. We present a focused systematic review of the literature on perioperative management in PH, and describe the development of a multidisciplinary perioperative best practice pathway for PH patients (PH Pathway) to address these challenges at a tertiary academic referral center.

## Review

Perioperative outcomes in patients with PH

PH is defined hemodynamically by a resting mean pulmonary arterial pressure greater than or equal to 25 mm Hg [3]. Elevations in PA pressure can arise as a consequence of increased pulmonary vascular resistance, cardiac output, and/or pulmonary artery wedge pressure (PAWP). Patients with PH are currently classified into five groups based on similarities in hemodynamics, pathophysiology, and therapeutic approach. The most recent classification, updated at the Fifth World Symposium [4], includes patients with pulmonary arterial hypertension (PAH) (Group I), patients with PH due to left heart disease (Group II), patients with PH due to chronic lung disease and/or hypoxia (Group III), patients with chronic thromboembolic PH (Group IV), and patients with PH due to unclear multifactorial mechanisms (Group V).

A number of retrospective analyses have demonstrated the considerable risk of morbidity and mortality in patients with PH undergoing non-cardiac surgery. A brief synopsis of these studies is presented in Table 1. There are important differences in the study methodology, the methods of defining PH (echocardiography vs. ICD-9 coding vs. right-heart catheterization), the classification of PH, and the era of data collection, all of which limits comparability among the studies. Despite these limitations, the reported mortality and morbidity among patients with PH is consistently high. Among well-characterized subjects with hemodynamically defined pre-capillary PH or PAH, mortality for non-cardiac surgery ranged from 3.5%-8% [5-7]. Where reported, death was most often due to right heart failure and generally occurred within 48 hours of the procedure. The types of surgery associated with higher mortality ranged from minor procedures, such as laparoscopic cholecystectomy, to major surgery (e.g., major bowel resection). Furthermore, emergency procedures were associated with a higher mortality risk. Patients with PAH were also found to be at higher risk compared with patients with other types of PH. Serious perioperative morbidities were also reported at rates of 24%-42%, with common complications including respiratory failure (7%-28%), congestive heart failure and/or volume overload (10%-13.5%), arrhythmia (12%), hemodynamic instability (8%), acute kidney injury (7%-10%), and myocardial ischemia (4%). In one study by Kaw, et al., important cost-related outcomes were also influenced by the presence of PH, with significantly longer intensive care unit (0.66 vs. 0.1 days, P = 0.04) and hospital length of stay (7 vs. 3.2 days, P = 0.0008), as well as a 2.4 fold increased risk of re-admission within 30 days that trended towards statistical significance [6]. This data was not broken down according to anesthesia type.

**Table 1:** Summary of studies of patients with pulmonary hypertension undergoing non-cardiac surgery, showing morbidity and mortality, as well as baseline characteristics PH: pulmonary hypertension; PAH: pulmonary arterial hypertension; NYHA: New York Heart Association; MPAP: mean pulmonary arterial pressure; GA: general anesthesia; SA: spinal anesthesia; IV: heavy sedation; EA: epidural anesthesia; RA: regional anesthesia; THR/TKR: Total hip/knee replacement; PHTN: pulmonary hypertension; PVH: pulmonary venous hypertension. *obtained through right heart catheterization

Study	Ramakrishna, et al. [8]	Lai, et al. [9]	Price, et al. [5]	Memtsoudis, et al. [10]	Kaw, et al. [6]	Meyer, et al. [7]
Number of subjects	145	62	28	3543	96	114
Type of study	Retrospective	Retrospective Controlled	Retrospective	NIS database Matched Samples	Retrospective Controlled	Prospective
Morbidity	42%	24%	29%	Not reported	28%	Not reported
Mortality	7%	9.7%	7%	2.4% / 2.9%	1%	3.5%
Mean Age (years)	60.1	67	53	74 (THR) / 71.7 (TKR)	62.4	57
Female Sex (%)	73	39	57	THR/TKR 68.4/71.7	50	70
PH Designation (%)	Group I (55) Group III (19) Group IV (8) Group V (19)	Group I (17.7) Group II (43.5) Group III (21) Group IV (3.2) Undetermined (14.5)	Group I (62) IPAH (36) Associated PAH (36) Group IV (28)	THR Primary (19.9) Secondary (80.1) TKR Primary (17.8) Secondary (82.2)	PAH (12.5) PVH (39.5) Mixed PH (48)	PAH (100)
Mean RVSP (mm Hg)	68 mm Hg	70 – 122		Not reported	49.4	Not reported
Mean MPAP* (mm Hg)	44	?	43	Not reported	37.3	45
NYHA class (%)	I (27) II (46) III/IV (27)	Not reported	I/II (75) III (25) IV (0)	Not reported	Not reported	I/II (54) III/IV (43)
Mean 6 min walk test (meters)	319	Not reported	388	Not reported	Not reported	399
PH vasodilator therapies (%)	Yes (14)	No	Yes (57)	Not reported	Not reported	Yes (100)
Surgery (%)	Low risk (21) Int/High risk (79)	Low risk (22) Int risk (40) High risk (0)	Minor risk (43) Major risk (57) Emergency (14)	THR (38) TKR (62)	Minor risk (35.4) Int risk (52.1) Major risk (12.5)	Elective / int risk (89) Emergency (11)
Anesthetic type (%)	GA (100)	GA (36%) SA (20%) IV (5%) EA (1%)	GA ± RA (50) RA (50)	Not reported	GA (100)	GA (82) SA (18)
Study limitations	75% patients class I/II No control ECHO data used to define PH	Doppler ECHO criteria	Mild to moderate disease No control	Limited clinical information including severity of disease or intraoperative course	Limited intraoperative course details	Small sample size Patients with well controlled PAH

Preoperative evaluation and management

Due to the increased risk of morbidity and mortality for patients with PH presenting for non-cardiac surgery, a perioperative evaluation is essential and should include a comprehensive assessment by a multidisciplinary team [8]. Unfortunately, the available evidence demonstrates significant heterogeneity regarding pre-operative risk factors associated with surgical morbidity and mortality in these patients. There are, however, some risk factors that have been repeatedly associated with adverse outcomes, some of which are modifiable, while others are not. Broadly speaking, those factors can be grouped into procedure-related factors and patient-related factors. Procedure-related factors are the need for emergency surgery [5, 7, 9-10], intermediate to high-risk surgery [5, 11], and prolonged surgery (> three hours) [5, 11]. Patient-related factors that were consistently identified with adverse outcomes include high American Society of Anesthesiologists (ASA) class and concomitant cardiovascular disease [6, 9]. Hemodynamics, New York Heart Association (NYHA) functional class, and exercise capacity (six-minute walking distance) were only inconsistently linked to increased perioperative risk, though this is likely due to differences in the methodologies used to identify and evaluate patients in these reports [6-7]. In a well-characterized cohort of PAH patients, a right atrial pressure greater than 7 mm Hg and a six-minute walking distance less than 399 meters were associated with increased morbidity and mortality [7]. Kaw, et al. demonstrated an association between mean pulmonary artery pressure and perioperative morbidity, and further associated fewer adverse outcomes among subjects with isolated post-capillary PH (PAWP > 15 mm Hg, PVR < 3 WU) when compared with subjects with pure pre-capillary PH (PAWP < 15 mm Hg, PVR > 3 WU) or mixed disease (PAWP > 15 mm Hg, PVR > 3 WU) [6]. In general, these data suggest that patients with a pulmonary vascular disease, reduced exercise capacity, and elevated right heart filling pressures may be at a higher risk for perioperative complications.

There is limited evidence supporting any specific pre-operative testing in order to predict outcomes in patients with PH undergoing non-cardiac surgery. In general, patients with known or suspected PH should undergo a preoperative evaluation that considers a number of factors, including etiology of PH, PH severity, functional status, co-morbid conditions, type of surgery, urgency of the procedure, and medication optimization. For patients with inadequately characterized PH, consultation with a PH specialist should be considered to help with disease classification, assessment of functional status/exercise capacity, and the need to obtain additional hemodynamic measurements. For patients with known and well-classified disease, pre-operative evaluation can focus on optimization of medical therapies (including vasodilators and diuretics), exercise capacity, and in some cases hemodynamics. This evaluation may support delaying a procedure until medical or rehabilitative intervention can improve hemodynamics or exercise capacity. Moreover, there are practical considerations related to medication planning, since established pulmonary vasodilator therapies should routinely continue through the perioperative period [12-13]. In some cases, established oral therapies may not be available (e.g., due to prolonged nothing by mouth (NPO) status or formulary restrictions), and alternative plans will need to be addressed. Parenteral therapies may require additional planning so that these complex delivery systems can be administered by providers and nurses with experience in their management. The pre-operative period provides an ideal opportunity for collaboration between anesthesiologists, surgeons, and PH specialists to uniquely tailor operative plans to balance each patient’s surgical needs, while effectively mitigating perioperative morbidity and mortality. Therefore, we are proposing some general guidelines for the pre-operative evaluation of PH patients in Table 2 and outline our specific approach below.

**Table 2:** Proposed evidence-based preoperative recommendations PH: pulmonary hypertension; 6MWD: six minute walk test; RHC: right heart catheterization.

Complete disease phenotyping by PH specialist
Medication review and optimization two weeks prior to surgery
Clinical examination, consider 6MWD +/- RHC within two weeks of surgery
Surgical planning, discussion to minimize operative time

Intraoperative management

Similar to pre-operative assessment, there are no comprehensive, evidence-based guidelines for the intraoperative management of patients with PH [8]. Strong evidence supporting any one specific anesthetic techniques or intraoperative intervention does not exist at the time of writing. There is some evidence that increased morbidity and mortality is associated with intra-operative vasopressor use [7, 11], though this association may be confounded by disease severity and with sicker patients requiring more interventions. An individualized plan based on the patient’s pathophysiology and comorbidities is critically important. It is imperative to maintain right ventricular function and to avoid inciting events that would cause pulmonary vasoconstriction (increase right ventricular afterload) or systemic hypotension (decrease right ventricular perfusion). A variety of anesthetic agents, none of which has been proven superior, can be used to accomplish these hemodynamic goals. In our hands, anesthetic options include managing an awake patient while providing analgesia by a regional or local technique, or general anesthesia with an advanced airway and controlled ventilation, which may also be supplemented by regional or local anesthesia techniques. Regardless of the anesthetic approach, selection of the best option for these patients involves minimizing intra-operative increases in pulmonary vascular resistance (Table 3). For example, combining a benzodiazepine with ketamine can block ketamine’s known effect on pulmonary vascular constriction by minimizing catecholamine release [14-16]. Pure alpha agonists to maintain blood pressure should be avoided due to their effects on the pulmonary circulation and norepinephrine is preferable to phenylephrine in the clinical setting [17-18]. Alternatively, vasoconstrictors that have limited effects on the pulmonary vasculature, such as vasopressin, are also preferable [17-20]. Indeed, in the hypotensive patient, low dose vasopressin restores coronary blood flow to the right ventricle by increasing systemic vascular resistance [21]. Low dose dobutamine, has been shown to similarly decrease pulmonary vascular resistance, while slightly improving cardiac output. However, dobutamine also causes systemic vasodilation which exacerbates systemic hypotension due to inhaled or intravenous anesthetics. Therefore, norepinephrine, with both vasopressor and inotropic properties, is frequently preferred intraoperatively and has evidence supporting its utility in models of acute right ventricular failure [22-24]. Large fluid bloused (especially of cold fluids) should be avoided to counteract hypotension as an increased preload worsens right ventricular oxygen consumption. Inhaled nitric oxide (or inhaled prostacyclins) can be employed to quickly lower right ventricular afterload in patients with severe PH and acute decompensation [24].

**Table 3:** Perioperative changes that increase pulmonary vascular resistance IV: intravenous; PEEP: positive end expiratory pressure.

Hypoxia & hypercarbia
Due to sedation, analgesia, poor mask, delayed intubation
Acidosis
Secondary to hypovolemia, infection, decreased cardiac output
Hypothermia
Caused by cold IV fluids or ambient temperature
Atelectasis and Hyperinflation
Tidal volume, PEEP
Catecholamine release
Pain, inadequate anesthesia, anxiety
Medications
Pure alpha agonists

Additionally, surgical technique, although not studied in patients with PH, has an impact on hemodynamics. Both the length of the procedure and the surgical approach will influence anesthesia planning and should be tailored to mitigate risk for each individual patient. In some cases, a more invasive approach (e.g., open laparotomy vs. laparoscopy for abdominal surgery) may be preferred, given the effects of high abdominal insufflation pressures on pulmonary vascular resistance (through altered respiratory mechanics and hypercapnia) and right ventricular preload [25-26].

Intraoperative monitoring is mainly dictated by the severity of the PH, the patient’s comorbidities, exercise tolerance, and the surgical procedure. Invasive arterial blood pressure monitoring has a relatively low rate of complications and can safely be placed in an awake patient under local anesthesia. This monitor provides immediate feedback on hemodynamic changes and can alert to early warning signs of cardiovascular decompensation. Central lines, especially pulmonary artery catheters, have a relatively high complication rate and are placed much less frequently as they have not been shown to consistently improve outcomes [27-28]. The main advantage of central venous catheterization is the ability to have reliable access in order to administer high dose vasoactive medications. If more advanced cardiopulmonary monitoring is required, and if appropriate equipment and personnel are available, trans-esophageal echocardiography (TEE) provides direct visualization of cardiac filling and function, as well as a means to calculate pulmonary pressures and cardiac output.

Postoperative management

Our literature review suggests that most complications occur during the postoperative period [5]. Vigilance to signs of a failing right ventricle, which is the major culprit of these complications, is key to detect and manage these patients. Post-operative planning should, therefore, be an important component of the pre-operative evaluation. In general, postoperative disposition depends on the severity of the patient’s disease and the complexity of the surgery. For example, patients with severe but compensated PH undergoing cataract surgery might be done on an outpatient basis, while the identical patient undergoing a craniotomy for a brain tumor warrants post-operative care in an intensive care unit (ICU). In some patients with severe PH, the experience of ICU staff in managing PH may outweigh the ICU experience in managing post-operative needs (e.g., medical vs. surgical ICU). Patients on parenteral therapy will benefit from post-operative care in a location staffed by providers and nurses experienced in the management of these complex medications, regardless of surgical risk. There are some data suggesting that patients with PAH benefit from having surgery in a center with experienced PH providers [7], and this approach has been advocated in recent guidelines [29].

Proposal of a PH pathway

**Figure 1:**
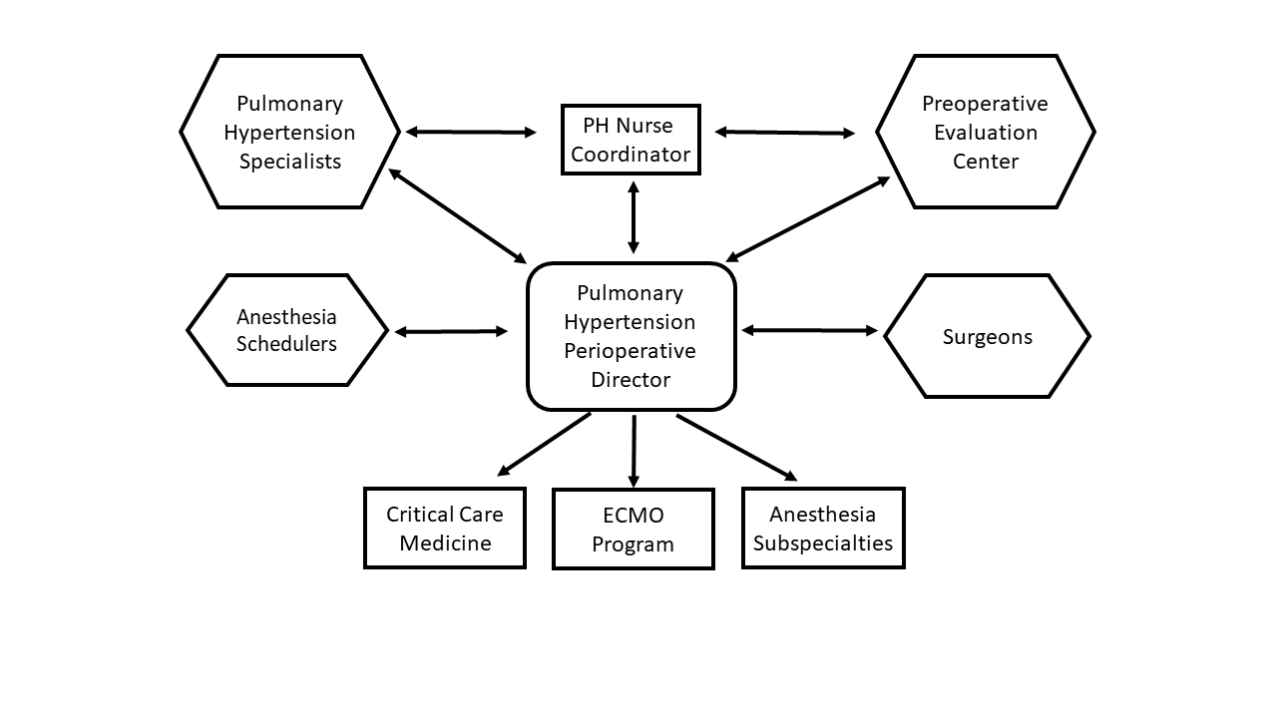
Program structure The pulmonary hypertension Perioperative Director receives consults and referrals from the Johns Hopkins Hypertension Program, the Preoperative Evaluation Center, the anesthesia scheduler, or the surgeons and coordinates perioperative care with all the stakeholders. ECMO: extracorporeal membrane oxygenation; PH: pulmonary hypertension

Given the increased risk of morbidity and mortality in PH patients presenting for non-cardiac surgery (1-2), and the lack of comprehensive guidelines for perioperative evaluation and management as presented above, our institution proposed a PH pathway that is centered around a comprehensive and multidisciplinary perioperative management team to evaluate all patients with PH presenting for non-cardiac surgery (Figure 1). Prior to implementation of this pathway, the evaluation and planning for patients with PH undergoing non-cardiac surgery at our institution were similar to that for patients without PH. Approximately 40% of surgical patients were seen in a pre-operative evaluation center (PEC) before the day of surgery. The remaining patients were evaluated by an anesthesiologist on the day of surgery. In some cases, procedures were cancelled on the day of surgery when severe PH was identified. Decisions regarding timing and location of surgical procedures, anesthesia staffing, and postoperative care were centered on surgical needs and operating room availability with limited emphasis on underlying PH-associated risk.

Key components of a PH pathway

Our multi-disciplinary approach implemented four specific interventions:

1. A cardiac anesthesiologist was designated Director of Perioperative Medicine for Adult patients with PH (PH Perioperative Director), serving as the liaison between medicine, surgery, and anesthesiology and coordinating perioperative care for those patients. Specific duties included handling referrals from the adult PH clinic, PEC, and surgeons, pre-operative assessment of PH patients, coordination of procedure timing, location, and staffing, intraoperative management recommendations, and decisions on post-operative destination.

2. For patients with previously undiagnosed PH, expedited review by providers in the PH clinic (electronic case review and/or prioritized clinic evaluation) was implemented to determine whether additional pre-operative evaluation or medical optimization was warranted.

3. Designated anesthesiology staffing for PH patients undergoing endoscopic procedures. We elected to provide staffing by a cardiac trained anesthesiologist for the endoscopy suite twice weekly.

4. Educational outreach to the Departments of Anesthesiology, Surgery, and Medicine was implemented to increase awareness of the unique risks in the perioperative management of this population.

Approach to perioperative management by the PH perioperative director

In an effort to integrate the available data on perioperative risk in PH patients, and in order to generate a standardized approach to pre-operative evaluation and planning, we charged the PH Perioperative Director with considering nine seminal questions for each PH patient (Table 4).

**Table 4:** Standardized pre-operative planning questions PH: pulmonary hypertension; ECMO: extra corporal membrane oxygenation.

Do the benefits of the surgery outweigh the PH-associated risks of the procedure?
Is the patient medically optimized?
How will PH medications be managed in the perioperative period?
Are procedural modifications necessary to mitigate PH-associated risk?
Should the procedure be moved from its usual location?
How should anesthesia staffing be allocated?
What is the optimal post-operative disposition?
Is the patient a candidate for ECMO?
Are there special circumstances requiring additional expert input?

1) Do the benefits of the surgery outweigh the PH-associated risks of the procedure? Anesthesiologists and PH specialists provide context for procedural risk in collaboration with the surgeon, in order to optimize risk-benefit calculations for each patient. Elective but necessary and urgent procedures may be carefully considered even in patients with the most severe hemodynamic derangements.

2) Is the PH patient medically optimized? In close collaboration with institutional PH specialists, and in some cases local cardiologists and internists, the PH Perioperative Director needs to determine if the patient is optimally treated with diuretics, pulmonary vasodilators, and therapies for other co-morbidities.

3) For PH patients on vasodilator therapy, how will medications be managed in the perioperative period? Abrupt discontinuation of pulmonary vasodilators can be associated with rebound PH and hemodynamic instability. The management of PH patients requires medical experts familiar with inhaled or parenteral prostacyclin therapy, their pharmacology and delivery systems, as even temporary interruptions can be life-threatening.

4) Are procedural modifications necessary to mitigate PH-associated risk? Utilizing local or regional anesthesia might obviate the need for mechanical ventilation. In other circumstances, a “more invasive” procedure may be appropriate if it mitigates PH-specific risk; e.g., open cholecystectomy instead of a laparoscopic procedure.

5) Should the procedure be moved from its usual location? We aim to bring necessary anesthesia resources to the scheduled operating environment (preserving local nursing and equipment expertise). In certain circumstances, procedures can be moved to the inpatient operating rooms (ORs), or to the cardiac ORs if specialized equipment/services are required.

6) How should anesthesia staffing be allocated? The PH Perioperative Director makes recommendations on optimal staffing for each case. This includes the number of cases and providers covered per anesthesiologist and the need for additional support from providers with unique expertise (e.g., regional anesthesia).

7) What is the optimal postoperative disposition? At our institution, the medical ICU is the preferred postoperative destination for high-risk PH patients undergoing minor surgical procedures. In the absence of a medical ICU bed, patients on inhaled or parenteral prostacyclin therapy should only be managed in select units with physician and nursing expertise in these therapies.

8) Is the patient a candidate for extracorporeal life support / extracorporeal membrane oxygenation (ECLS/ECMO)? This question should be addressed preoperatively. In some cases, ECMO could be used as a bridge to recovery (or transplantation) in the setting of perioperative hemodynamic collapse. The complexities of this decision mandate discussion prior to the case, not on an emergency basis.

9) Are there any special circumstances to be taken in consideration (e.g., obstetric care)? PH is associated with high peri-partum mortality [30] and presents a unique challenge. While a debate exists between the relative benefits of natural labor vs. planned surgical (cesarean) delivery, recent expert consensus recommendations favor the latter [31]. In these circumstances, maternal-fetal medicine and obstetrical anesthesiology experts should be engaged to help coordinate and provide perioperative care.

Case series

In an effort to evaluate the impact of implementing the PH pathway on workflow, we performed a retrospective analysis of cardiac anesthesia involvement in all subjects in the institutional review board (IRB) approved Johns Hopkins PH Registry (NA_00027124) who underwent non-cardiac surgery or endoscopy in the two-year period surrounding the implementation of the pathway. This study was evaluated by the Johns Hopkins IRB as Quality Improvement Project (IRB00117581) and it was determined that it does not constitute human subjects research under the Department of Health and Human Services (DHHS) or Federal Drug Administration (FDA) regulations given the retrospective nature. We hypothesized that implementation of the pathway increased cardiac anesthesiology involvement in these high-risk cases. We further evaluated some crude outcome measures as a preliminary metric of the benefits of this pathway for patients. We identified 38 patients who underwent 56 different procedures requiring anesthesia or moderate sedation (Table 5). Implementation of the PH pathway was associated with an increase in cardiac anesthesia involvement in surgical procedures (79% vs. 54%; P = 0.09). Cardiac anesthesia involvement increased in both endoscopic (64% vs. 38%; P = 0.43) and operating room (93% vs. 67%; P = 0.17) procedures, though the numbers were too small to demonstrate statistical significance. During the time frame included in the analysis, there were 26 unique hospitalizations associated with 34 inpatient procedures. There was no difference in mean (16.5 vs. 16.3 days; P = 0.97) or median (16 vs. 10 days; P = 0.88) hospital length of stay after implementation of the PH pathway. However, 30-day readmission rates among these patients decreased from 50% to 0% (P = 0.003). In the 22 outpatient procedures, there was no difference in 30-day readmission between procedures performed before (0/9, 0%) and after (2/13, 15%) implementation of the PH pathway (P = 0.49).

**Table 5:** Baseline demographic data for patients with pulmonary hypertension prior to and following implementation of the PH pathway *Total unique patients = 38; 2 had procedures after the intervention. WHO: World Health Organization; Endo: endoscopy; Prost: prostacyclin therapy; NYHA: New York Heart Association; FC: functional class; CA: cardiac anesthesia; LOS: length of stay; PH: pulmonary hypertension.

	Pre-PH Pathway	Post-PH Pathway	P-value	
Unique patients*	18	22		
WHO Group I, N (%)	12 (67%)	14 (64%)		1.0
Female sex, N (%)	17 (94%)	18 (82%)		0.36
Total procedures	28	28		1.0
Endo, N (%)	13 (48%)	14 (50%)		1.0
OR, N (%)	15 (54%)	14 (50%)		1.0
Med. Age at Surg, y (Min, Max)	59 (37, 74)	62 (35, 87)		0.01
NYHA FC at Surg				1.0
I-2, N (%)	15 (54%)	16 (57%)		
3, N (%)	13 (46%)	12 (43%)		
PH Therapy at Surg				
Oral, N (%)	23 (82%)	25 (89%)		0.71
Prostacylin, N (%)	14 (50%)	7 (25%)		0.10
Oral/Prost Combo, N (%)	11 (39%)	6 (21%)		0.24
CA involved, N (%)	15 (54%)	22 (79%)		0.09
Inpatient procedures, N	19	15		
Unique Hospitalizations, N	12	14		
Mean LOS, d (SD)	16.8 (13.9)	15.7 (13.2)		0.92
Median LOS, d (Min, Max)	12 (3, 38)	15 (2, 44)		0.84
Readmission 30-d, N (%)	6 (50%)	0 (0)%		0.003

## Conclusions

Patients with PH presenting for non-cardiac surgery are at an increased risk of morbidity and mortality. Advances in treatment have resulted in improved survival and more patients are presenting for non-cardiac surgery. In order to mitigate risk and improve outcome, a multidisciplinary team approach can be implemented pre-operatively to coordinate pre-operative evaluation, intra-operative management, and post-operative care. Our institution developed a multidisciplinary team-based approach for the perioperative evaluation of patients with pulmonary hypertension. Our preliminary data obtained before and after implementation of the perioperative clinical PH pathway suggests that these interventions increased pathway utilization and reduced readmission rates for hospitalized patients undergoing surgery. Although this analysis has methodological limitations (small sample size, retrospective design), the findings represent the successful implementation of the PH pathway and provide a justification for prospective analyses designed to further delineate “best practice” for the perioperative management of this patient cohort. A strength of the described approach is the alignment with recent guidelines recommending multidisciplinary surgical care at a PH center and the concept of a Perioperative Surgical Home model. While this specific model may only be applicable to a tertiary academic center with multiple sub-specialties, other iterations of this approach could be applicable in other settings. Implementation of a standardized planning and evaluation program, as outlined above, will facilitate the early identification of these patients, allowing for the optimization of staffing and resource allocation at the local institution, or referral to a tertiary center if more specific expertise is required.

One key question raised from our experience is, should a cardiac anesthesiologist be required for every PH patient, as suggested by recent management guidelines? We posit that most of the benefits that we have seen from this program are a result of the organization and communication involved in each individual patient considered through a multi-disciplinary approach in advance of surgery, rather than from the specific medical knowledge or skills possessed by the designated anesthesiologists. In our institution we routinely have a cardiac anesthesiologist participate in the intra-operative care if the likelihood of perioperative instability is felt to be high, or if advanced monitoring (transesophageal echocardiography) is planned, and/or if ECMO backup has been arranged.

The perioperative management of PH patients is complex, and evidence-based guidelines do not exist. We have developed a multi-disciplinary approach to planning, with specific interventions and pre-operative considerations. Overall, the development of this multidisciplinary program has been met with positive feedback from our patients, surgical colleagues, and hospital administration, and should provide a framework for future studies designed to identify and mitigate unique perioperative risks in these patients.

## References

[1] Badesch DB, Raskob GE, Elliott CG (2010). Pulmonary arterial hypertension: baseline characteristics from the REVEAL Registry. Chest.

[2] Humbert M, Sitbon O, Chaouat A (2010). Survival in patients with idiopathic, familial, and anorexigen-associated pulmonary arterial hypertension in the modern management era. Circulation.

[3] Hoeper MM, Bogaard HJ, Condliffe R (2013). Definitions and diagnosis of pulmonary hypertension. J Am Coll Cardiol.

[4] Simonneau G, Gatzoulis MA, Adatia I (2013). Updated clinical classification of pulmonary hypertension. J Am Coll Cardiol.

[5] Price LC, Montani D, Jais X, Dick JR, Simonneau G, Sitbon O, Mercier FJ, Humbert M (2010). Noncardiothoracic nonobstetric surgery in mild-to-moderate pulmonary hypertension. Eur Respir J.

[6] Kaw R, Pasupuleti V, Deshpande A, Hamieh T, Walker E, Minai OA (2011). Pulmonary hypertension: an important predictor of outcomes in patients undergoing non-cardiac surgery. Respir Med.

[7] Meyer S, McLaughlin VV, Seyfarth HJ (2013). Outcomes of noncardiac, nonobstetric surgery in patients with PAH: an international prospective survey. Eur Respir J.

[8] Ramakrishna G, Sprung J, Ravi BS, Chandrasekaran K, McGoon MD (2005). Impact of pulmonary hypertension on the outcomes of noncardiac surgery: predictors of perioperative morbidity and mortality. J Am Coll Cardiol.

[9] Lai HC, Wang KY, Lee WL, Ting CT, Liu TJ (2007). Severe pulmonary hypertension complicates postoperative outcome of non-cardiac surgery. Br J Anaesth.

[10] Memtsoudis SG, Ma Y, Chiu YL, Walz JM, Voswinckel R, Mazumdar M (2010). Perioperative mortality in patients with pulmonary hypertension undergoing major joint replacement. Anesth Analg.

[11] Yang EI (2015). Perioperative management of patients with pulmonary hypertension for non-cardiac surgery. Curr Rheumatol Rep.

[12] Fox DL, Stream AR, Bull T (2014). Perioperative management of the patient with pulmonary hypertension. Semin Cardiothorac Vasc Anesth.

[13] Tonelli AR, Minai OA (2014). Saudi guidelines on the diagnosis and treatment of pulmonary hypertension: perioperative management in patients with pulmonary hypertension. Ann Thorac Med.

[14] Hatano S, Keane DM, Boggs RE (1976). Diazepam-ketamine anaesthesia for open heart surgery a "micro-mini" drip administration technique. Can Anaesth Soc J.

[15] Jackson AP, Dhadphale PR, Callaghan ML, Alseri S (1978). Haemodynamic studies during induction of anaesthesia for open-heart surgery using diazepam and ketamine. Br J Anaesth.

[16] Kumar SM, Kothary SP, Zsigmond EK (1978). Plasma free norepinephrine and epinephrine concentrations following diazepam-ketamine induction in patients undergoing cardiac surgery. Acta Anaesthesiol Scand.

[17] Wang M, Shibamoto T, Kuda Y, Tanida M, Kurata Y (2015). Systemic vasoconstriction modulates the responses of pulmonary vasculature and airway to vasoconstrictors in anesthetized rats. Exp Lung Res.

[18] Kwak YL, Lee CS, Park YH, Hong YW (2002). The effect of phenylephrine and norepinephrine in patients with chronic pulmonary hypertension. Anaesthesia.

[19] Trempy GA, Nyhan DP, Murray PA (1994). Pulmonary vasoregulation by arginine vasopressin in conscious, halothane-anesthetized, and pentobarbital-anesthetized dogs with increased vasomotor tone. Anesthesiology.

[20] Evora PR, Pearson PJ, Schaff HV (1993). Arginine vasopressin induces endothelium-dependent vasodilatation of the pulmonary artery. V1-receptor-mediated production of nitric oxide. Chest.

[21] Tsuneyoshi I, Onomoto M, Yonetani A, Kanmura Y (2005). Low-dose vasopressin infusion in patients with severe vasodilatory hypotension after prolonged hemorrhage during general anesthesia. J Anesth.

[22] Kerbaul F, Rondelet B, Motte S (2004). Effects of norepinephrine and dobutamine on pressure load-induced right ventricular failure. Crit Care Med.

[23] Brown NJ, Bradford J, Wang Z (2007). Modulation of angiotensin II and norepinephrine-induced plasminogen activator inhibitor-1 expression by AT1a receptor deficiency. Kidney Int.

[24] Lahm T, McCaslin CA, Wozniak TC (2010). Medical and surgical treatment of acute right ventricular failure. J Am Coll Cardiol.

[25] Blaney MW, Calton WC, North JH, Jr Jr (1999). The effects of preperitoneal carbon dioxide insufflation on cardiopulmonary function in pigs. JSLS.

[26] Liem T, Applebaum H, Herzberger B (1994). Hemodynamic and ventilatory effects of abdominal CO2 insufflation at various pressures in the young swine. J Pediatr Surg.

[27] Gidwani UK, Goel S (2016). The pulmonary artery catheter in 2015: the swan and the phoenix. Cardiol Rev.

[28] Gidwani UK, Mohanty B, Chatterjee K (2013). The pulmonary artery catheter: a critical reappraisal. Cardiol Clin.

[29] Taichman DB, Ornelas J, Chung L (2014). Pharmacologic therapy for pulmonary arterial hypertension in adults: CHEST guideline and expert panel report. Chest.

[30] Jais X, Olsson KM, Barbera JA (2012). Pregnancy outcomes in pulmonary arterial hypertension in the modern management era. Eur Respir J.

[31] Hemnes AR, Kiely DG, Cockrill BA (2015). Statement on pregnancy in pulmonary hypertension from the Pulmonary Vascular Research Institute. Pulm Circ.

